# Differentially Expressed Proteins in Malignant and Benign Adrenocortical Tumors

**DOI:** 10.1371/journal.pone.0087951

**Published:** 2014-02-03

**Authors:** Hanna Kjellin, Henrik Johansson, Anders Höög, Janne Lehtiö, Per-Johan Jakobsson, Magnus Kjellman

**Affiliations:** 1 Rheumatology Unit, Department of Medicine, Karolinska Institutet, Stockholm, Sweden; 2 Department of Molecular Medicine and Surgery, Karolinska Institutet, Stockholm, Sweden; 3 Department of Oncology-Pathology, Karolinska Institutet, Stockholm, Sweden; 4 Science for Life Laboratory, Cancer Proteomics Mass Spectrometry, Karolinska Institutet, Solna, Sweden; 5 Department of Breast- and Endocrine Surgery, Section of Endocrine and Sarcoma Surgery, Karolinska University Hospital, Stockholm, Sweden; Universidade Federal do Rio de Janeiro, Brazil

## Abstract

We have compared the microsomal protein composition of eight malignant and six benign adrenocortical tumors with proteomic methods. IGF2 had increased level in the malignant tumors, confirming previous microarray studies on the same material. Aldolase A, a glycolytic enzyme, also showed increased levels in the malignant tissue compared to the benign. Additionally, several proteins belonging to complex I in the mitochondrial respiration chain showed decreased levels in the malignant tissue. Taken together, this may indicate a shift in energy metabolism where glycolysis may be favored over tight coupling of glycolysis and mitochondrial respiration, a phenomenon known as the Warburg effect. One of the complex I proteins that showed decreased levels in the malignant tissue was GRIM-19. This protein has been suggested as a tumor suppressive protein by being a negative regulator of STAT3. In summary, an analysis of the microsomal proteome in adrenocortical tumors identifies groups of proteins as well as specific proteins differentially expressed in the benign and malignant forms. These proteins shed light on the biology behind malignancy and could delineate future drug targets.

## Introduction

Adrenocortical tumors have a relatively high prevalence in the general population of up to 9% in autopsy studies [1]. However, malignancies are rare, with a yearly incidence of 2 per million inhabitants, but they have a poor prognosis [2]. Recent advances in the bioimaging field together with the more frequent use of computed tomography (CT) and magnetic resonance imaging (MRI) have increased the number of detected adrenocortical tumors [3]. These incidentally discovered tumors are called adrenal incidentalomas and the majority of them are benign and non-functioning adenomas [4]. Distinguishing between adrenocortical carcinomas (ACCs) and adrenocortical adenomas (ACAs) can be difficult. In the clinical decision making, the tumor size and the CT Hounsfield measurements are the most important features in determining if the tissue alteration is benign or malignant. Masses less than 3 cm in diameter are usually benign; by contrast, if the mass is larger than 6 cm the probability of malignancy increases [5]. Masses measuring between 3 and 6 cm are uncertain and since early resection of ACCs is the best chance of survival, an accurate diagnosis of a small tumor is very important [5]. All tumors with a diameter larger than 4 cm are recommended to be resected [6]. There is therefore a need for improved diagnostic biomarkers, especially to avoid unnecessary abdominal surgery.

The aim of this study was to shed light on the molecular pathology behind the malignant phenotype of ACCs. To increase the analytical depth of our analysis, we fractionated the whole cell lysate generated from the tissue homogenisation. We chose to enrich for the microsomal protein fraction, since this fraction will consist of both soluble and membrane-bound proteins and could therefore contain interesting molecules involved in cancer signalling networks. Using this enrichment strategy could also increase the chances of identifying potential membrane markers that can be used to differentiate between ACAs and ACCs. Herein, an analysis of the microsomal proteome in adrenocortical tumors identified groups of proteins as well as specific proteins differentially expressed in ACAs and ACCs. We specifically observed changes in several mitochondrial proteins, with emphasis on pathways related to energy metabolism. These findings will be discussed regarding their potential role in adrenocortical tumorigenesis.

## Results and Discussion

**Figure 1:**
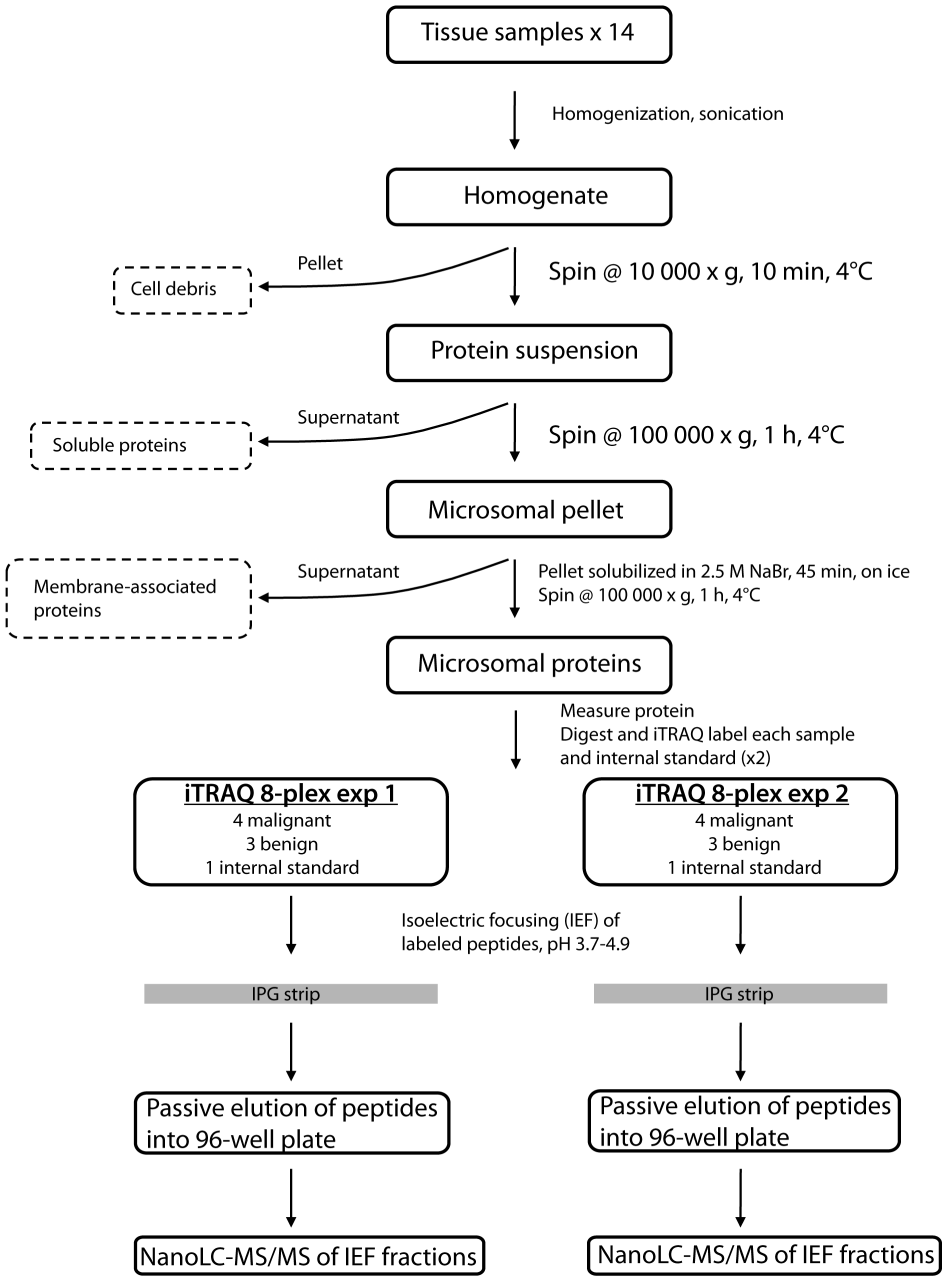
Illustration of the experimental procedure. The 14 samples (eight ACCs and six ACAs) were run in two separate 8-plex iTRAQ sets. An internal standard was included in each iTRAQ set. See Experimental procedures for details.

In this study we wanted to perform a proteomic analysis to shed light on the molecular pathology behind the malignancy phenotype of ACCs. To reduce the complexity of the sample we enriched for microsomal proteins. The method used includes ultracentrifugation for enrichment of microsomal proteins, tryptic digestion, iTRAQ (isobaric Tag for Relative and Absolute Quantification) labeling of the peptides followed by a two-dimensional separation strategy using narrow range isoelectric focusing (IEF) and reversed phase liquid chromatography, and finally Orbitrap tandem mass spectrometry (MS) for identification of the proteins (Fig. 1). The IEF step yields 72 peptide fractions and for this study, 42 of these were analyzed by liquid chromatography (LC)-MS/MS and approximately 3300 proteins were identified with a false discovery rate (FDR) of <1% (Tables S1, S5 and S6). For all patients, 1902 proteins could be accurately quantified with at least one 99% confident peptide (Table S2).

### Enrichment analysis

**Figure 2:**
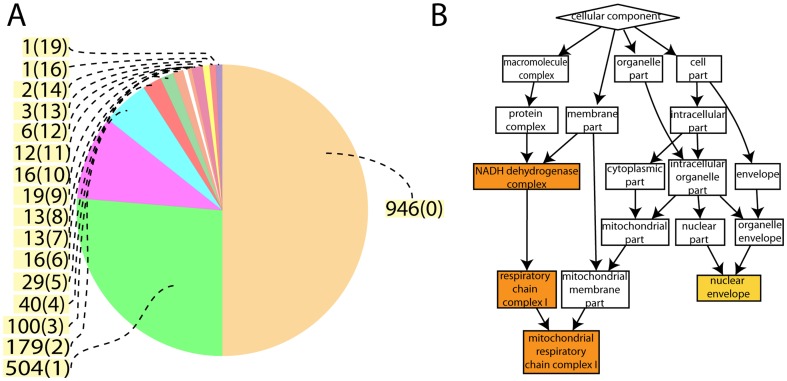
Enrichment analysis. Panel A shows a pie chart depicting the number of transmembrane segments the 1902 identified and quantified proteins are predicted to contain (from ProteinCenter). Panel B shows the GOrilla results performed on the 1902 proteins, ranked by t-test p-value. Shown are enriched GO terms in “cellular component”.

The 1902 identified and quantified proteins were analyzed with ProteinCenter. An over-representation of the membrane protein domain was observed (p = 9.13×10^−7^), using the human genome as a background. Most membrane proteins were predicted to have one transmembrane segment but some as many as 19 transmembrane segments, Fig. 2A. GOrilla [7], [8], a software to search for enrichment of gene ontology (GO) terms, indicated enrichment of mitochondrial proteins within the term “cellular component” (single ranked list, sorted on p-value from student's t-test), Fig. 2B. These analyses show that we have succeeded in enriching for microsomal proteins, since microsomes should contain proteins both from different organelle membranes as well as soluble proteins with their function close to these organelles.

### Statistics

**Figure 3:**
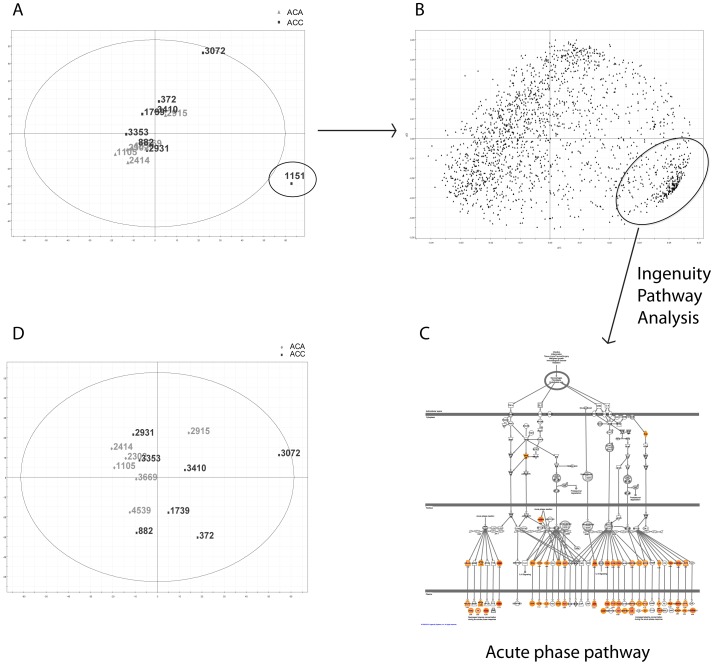
Multivariate data analysis and outlier detection. Panel A shows a PCA plot of the data. Sample 1151 was identified as an outlier. Panel B shows the underlying data that formed the PCA in panel A. Circled are the proteins causing the deviating behavior of sample 1151. These proteins were run through the Ingenuity Pathway Analysis program. In panel C the top canonical pathway is shown; acute phase pathway. A PCA without sample 1151 is shown in panel D; no more outliers were found.

**Figure 4:**
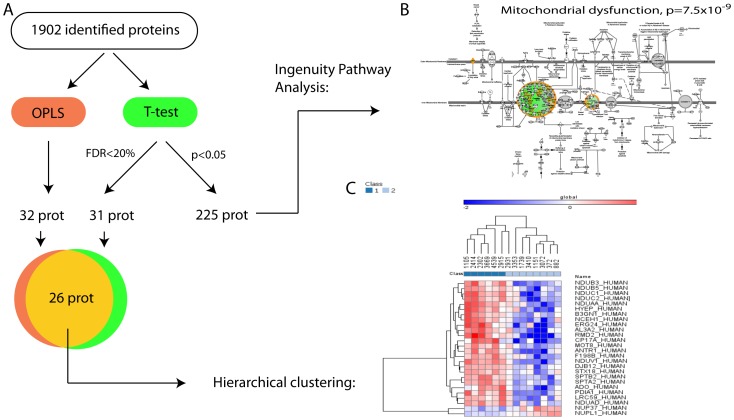
Visualization of the data analysis workflow. A) Both univariate (Student's t-test) and multivariate (OPLS) analyses were performed. B) The top canonical pathway identified by Ingenuity Pathway Analysis was mitochondrial dysfunction. Many proteins in this pathway were found to be downregulated in the malignant samples. C) Hierarchical clustering of the overlapping proteins (t-test and OPLS). Class 1: ACA, class 2: ACC.

To verify that the patients have a representative quantitative ratio distribution and to identify potential biases between patients, we used Simca-P+ 12.0 to perform a principal component analysis (PCA). Fig. 3A shows the generated PCA plot, where sample 1151 is identified as an outlier. To investigate the reason for this, the loadings (proteins) contributing to the deviating behavior of this sample were analyzed with the Ingenuity pathway analysis (IPA) program (Fig. 3B–C). The top canonical pathway identified was the acute phase pathway (Fig. 3C). This fact, together with visual inspection of the sample, led us to speculate that the reason for sample 1151 being an outlier was plasma protein (blood) contamination. A new PCA was performed without sample 1151 and no more outliers could be identified (Fig. 3D). Since we suspected blood contamination to be the reason for sample 1151 being an outlier, it was again included in the analyses that followed, but care was taken in the next steps of data analysis to ensure that sample 1151 did not skew the results. To identify proteins that could differentiate between ACAs and ACCs, orthogonal projections to latent structures (OPLS) was performed. In order to refine the generated OPLS model, proteins that had a small influence were removed. This was done by using the Variable Importance on Projection (VIP) scores. A final model of 32 proteins was created (Q = 0.859, p = 2.1×10^−5^). In parallel, a model was created where sample 1151 was excluded. No significant difference between the models was found (data not shown). Next, a student's t-test analysis was performed to identify individual proteins that differentiate ACAs from ACCs. With a FDR of 20%, 31 proteins were identified as up- or downregulated. The overlap between the OPLS model and the t-test was 26 proteins, Fig. 4A, and Table S3.

### Pathway analysis

Pathway analysis was performed on all proteins that were identified as differentially expressed by the t-test, using p<0.05 as a cutoff (225 proteins). The top canonical pathway was mitochondrial dysfunction (ratio 0.104, p = 7.5×10^−9^), Fig. 4B. All proteins (n = 14) that were found belonging to this pathway were downregulated and most of them belonged to complex I of the respiratory chain.

### Hierarchical clustering

The proteins that overlapped in the univariate and multivariate data analyses were subjected to hierarchical clustering. As can be seen in Fig. 4C, one of the malignant samples (2931) cluster together with the benign samples. The same was observed in a microarray study where that sample was included [9]. Also, only three Weiss histological criteria for adrenocortical cancer were fulfilled for this sample (necrosis, atypical mitosis and nuclear atypia) and the patient has not revealed metastasis, indicating uncertain diagnosis.

### Differentially expressed proteins

IGF2 (insulin-like growth factor 2) had increased levels in ACCs (fold: 6.5, p = 0.008). This is in agreement with several gene expression profiling studies on adrenocortical tumors [9]–[12]. The IGF signaling pathway plays a key role in malignant transformation and cancer progression and is also of interest as a target for cancer therapy (see e.g. [13]). Plasma levels of IGF binding protein 2 (IGFBP2), which in its phosphorylated form can regulate localization and translation of IGF2 mRNA [14], has been evaluated as a diagnostic marker in adrenocortical tumors, but it was not considered sensitive enough to be used clinically [15]. Nevertheless, our data confirms previous results regarding overexpresison of IGF2 expression in ACC.

**Figure 5:**
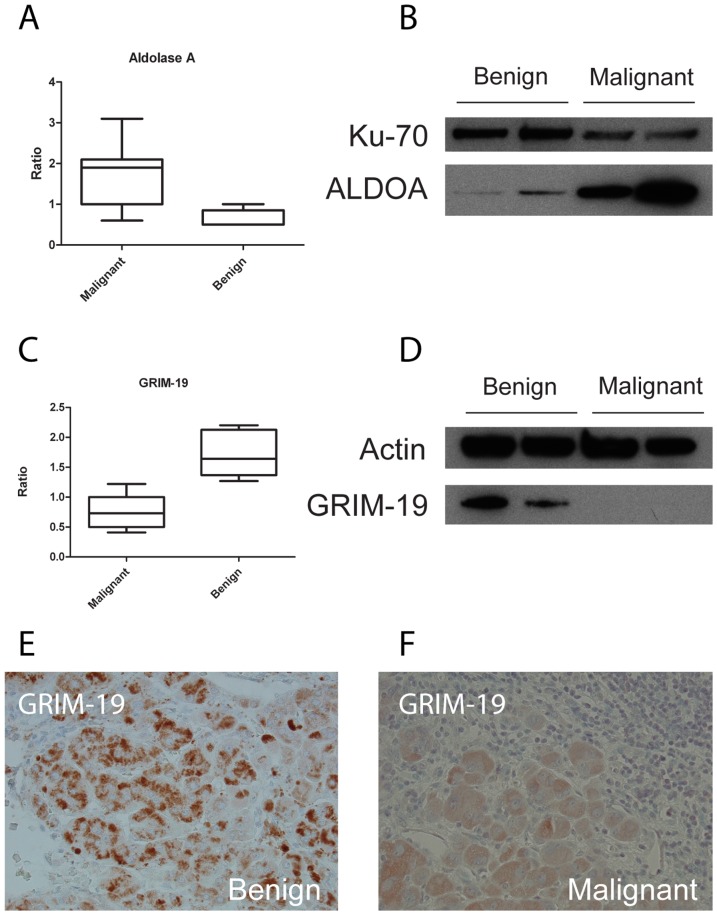
Western blot and immunohistochemical analyses. A–D) Expression levels of aldolase A and GRIM-19. To the left are the expression levels from the MS data and to the right are the western blot analyses. Ku70 and actin are loading controls. Western blot analyses were performed on tissue samples that showed the most significant differences in the iTRAQ experiments (two ACAs and two ACCs). E–F) Immunohistochemical analyses with anti-GRIM-19 (panels E and F). GRIM-19 staining in ACAs had a grain-like pattern, suggesting mitochondrial localization (panel E). In ACCs there was a more cytoplasmic staining (panel F).

Pathway analysis revealed that several proteins of complex I in the mitochondrial respiratory chain had decreased levels in ACCs. One of these was NDUFA13 (fold: 0.5, p = 0.0005), which was confirmed with western blot analysis on the same samples (Fig. 5D). NDUFA13, or GRIM-19 **(G**ene associated with **R**etinoic- and **I**nterferon-induced **M**ortality-19) has been identified as an IFN-β- and RA-induced gene with pro-apoptotic function in breast cancer cell lines [16]. The cellular localization of GRIM-19 is not completely demonstrated. It has been found to co-purify with mitochondrial complex I in both bovine and human heart [17], [18], and blue native gel electrophoresis (BN-PAGE) separation of mouse mitochondrial complex I with subsequent western blot analyses with antibodies against GRIM-19 confirmed localization in murine mitochondria [19]. In MCF-7 cells it has been found localized in both mitochondria and nucleus and it was postulated that this ambiguity may be due to different isoforms of the protein or that post-translational modifications regulate the cellular localization [20]. However, it has also been suggested that nuclear staining of GRIM-19 could be due to non-specific reactions and that the primary localization of the protein is in the mitochondria [19]. When bound to the mitochondrial membrane, GRIM-19 is involved in complex I assembly and activity and is required for electron transfer activity [21].

To further study GRIM-19 expression, three benign tissues and three malignant tissues were analyzed with immunohistochemical methods. In two of the benign samples there were indications of grain-like expression patterns (Fig. 5E and Fig. S1). On the contrary, in the malignant samples there was a more general cytoplasmic staining pattern (Fig. 5F). This could suggest a mitochondrial localization of GRIM-19 in ACAs and that this localization may have been lost in the ACCs, either due to loss of the protein or loss of functional protein. GRIM-19 has been shown to interact with STAT3 in various cell types [20], [22], [23]. STATs (signal transducers and activators of transcription) are a family of latent cytoplasmic transcription factors which are activated by binding to cytokine receptors and subsequent phosphorylation by the Janus kinases (JAKs) and play a role biological responses such as cell growth and apoptosis [24]. We examined two of the benign and two of the malignant tissues by immunohistochemistry but could not observe any obvious differences concerning the level of STAT3 expression (Fig. S1). GRIM-19 may interact with and regulate activated STAT3, as suggested in [25], and therefore it would be necessary to investigate the levels of phosphorylated STAT3 in these tissues. The role of GRIM-19 in cancer development has been reviewed in [26].

In our mass spectrometry data we did not see a complete loss of GRIM-19 expression, but a ∼2-fold difference in expression when comparing ACAs and ACCs. The identification and quantification of GRIM-19 was based on 2 peptides, one at position 61–68 and the other at position 70–81 (data not shown). Interestingly, western blot analyses could not detect any GRIM-19 at all in the malignant tissue (Fig. 5D). This could of course be due to sensitivity issues of the anti-GRIM-19 antibody or that the protein is completely lost but it could also suggest a defective protein, as suggested in [27]. The antibody used in our study recognizes the full-length protein and the reason for the absent signal in the western blot analyses could be inability of the antibody to recognize any modified form of the protein.

Aldolase A was identified as upregulated in the iTRAQ experiment (fold: 2.6, p = 0.003), which was confirmed by western blot analysis (Fig. 5B). Aldolase A is a glycolytic enzyme responsible for the cleavage of the six-carbon sugar fructose 1,6-biphosphate into the two three-carbon fragments glyceraldehyde 3-phosphate (GAP) and dihydroxyacetone phosphate (DHAP). The fact that cancer cells have increased glucose consumption, an effect of increased glycolysis, is undisputable and the phenomenon is readily used in the clinic with the application of the imaging technique positron-emission tomography (PET) using the glucose analogue tracer ^18^fluorodeoxyglucose (FdG). The phenomenon, that cancer cells predominantly produce energy by a high rate of glycolysis followed by lactic acid fermentation in the cytosol, rather than by a comparatively low rate of glycolysis followed by oxidation of pyruvate in mitochondria like most normal cells, is known as the Warburg effect [28], [29]. Warburg postulated in his original article that the reason for aerobic glycolysis was damaged respiration [28], [29], but later studies have shown that aerobic glycolysis is not unique to tumor cells, but also proliferating lymphocytes revealed a similar phenotype, and that it could instead be an effect of fast proliferating cells [30].

Interestingly, there is a link between activation of growth factor receptors and glycolysis, where IGF2 could play a role. Activation of growth factor receptors leads to PI3K activation, which via AKT leads to increased glucose uptake and flux through the early part of glycolysis [30], [31]. Additionally, tyrosine kinases, which many oncogenes are, can inhibit the later part of glycolysis through regulation of the M2 isoform of pyruvate kinase [32], and thereby intermediates of glycolysis can be used for amino acid and nucleotide synthesis. If the ACCs examined in this study have increased glycolysis rate, it could be due to a combination of impaired mitochondrial function, due to down-regulation or loss of functional GRIM-19, and activation of growth factor receptors through IGF2 signaling. Whether the ACCs in the current study have increased glycolysis rate or not needs to be further investigated by functional studies.

### Protein expression levels correlate to tumor size

**Figure 6:**
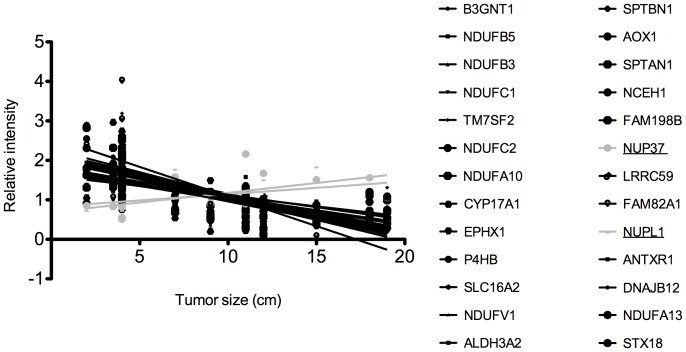
Correlation between protein expression levels and tumor size. Protein expression levels of the 26 proteins that overlapped in the t-test and OPLS analyses correlate with the size of the tumors. Two proteins have increased expression levels (light grey dots/lines), the rest have decreased expression levels (black dots/lines). Corresponding protein names can be found in Table S4.

As can be seen in Fig. 6, expression levels of the 26 proteins that overlapped in the univariate and multivariate analyses correlated with the tumor size; the larger the tumor, the higher expression of the up-regulated proteins and the lower the expression of the down-regulated proteins. This is interesting since the size of the tumor is one of the most important features when diagnosing the mass in the clinic [5]. Other studies have shown that the genetic instability increases with increased tumor size [33], [34], which could be reflected by the lowered expression levels of the down-regulated proteins. Additionally, a larger tumor could lead to increased hypoxia, which could shift the metabolism and favor glycolysis, and this is then reflected by decreased expression of several complex I proteins and increased expression of Aldolase A. As mentioned, the size of the tumor is important when deciding if to take out the tumor or not and today the threshold is set at 4 cm. However, retrospective studies on surgery performed on adrenal incidentalomas, show that only 10–16% of the resected tumors were in fact malignant [35], [36]. It was also shown that only 5% of resected tumors that were <3 cm were malignant. This means that size as a diagnostic marker has high sensitivity but low specificity. Future studies should aim at analyzing the protein expression in small ACCs to investigate if the protein expression pattern differs from ACAs of the same size. Unfortunately, the sample size of this study was too small to do this analysis.

## Conclusions

In summary, by analyzing the microsomal protein composition of ACAs and ACCs, we observe changes in the mitochondrial proteome including a reduced expression of GRIM-19 in ACCs. It is not clear at this point if it is a down-regulation *per se* or a loss of functional GRIM-19, however indications point towards a re-localization of the protein, from mitochondria to the cytoplasm, and this re-localization could be due to a modified form of the protein in ACCs. Studies are on-going to confirm this hypothesis. Re-localization of GRIM-19 could lead to dysfunctional mitochondria and STAT3 hyperactivation, which in turn could drive the tumorigenesis in ACCs. Further, we observe an indication of increased glycolysis rate in ACCs. However, the reason for the shift in metabolism is most probably multi-factorial, where e.g. hypoxia as well as increased expression of growth factors (IGF2) could play a role.

## Methods

### Ethics statement

The samples were obtained with verbal informed consent and the study of the tissue material was approved by the Ethical Committee (Dnr 01-136, 01-242) of the Karolinska University Hospital. All patients at the Department of Endocrine Surgery are asked before surgery if they allow tumor tissue and blood to be saved in the biobank at Karolinska Institutet for basic research purposes. The patient's decision is always noted in the pathological report. This routine has been approved for all types of tumor tissues operated at Karolinska University Hospital.

### Tissue samples

The tumor tissue analyzed contained more than 70% tumor cells. All patients had been operated on at Karolinska University Hospital and had a clinical follow-up for at least 1.5 years. Diagnostic criteria for malignancy were vascular invasion, invasion of surrounding organs and/or presence of distant metastasis. In cases where these criteria were not fulfilled the histopathological criteria proposed by Weiss was used [37]. Cases with high nuclear grade, increased mitotic figures and tumor necrosis were suspected to be malignant. The appearance of metastasis at follow-up, or recurrences further facilitated the establishment of a malignant diagnosis. The clinical and tumor characteristics are shown in table 1.

**Table 1:** Clinical data of analyzed cases.

Case No	Histopathological diagnosis	Sex	Age	Met. status	Size (cm)	Survival (months)^a^
1105	Adrenocortical adenoma	F	29		2	205
2302	Adrenocortical adenoma	F	64		4	150
2414	Adrenocortical adenoma	F	63		4	145
3669	Adrenocortical adenoma	F	54		4	85
2915	Adrenocortical adenoma	M	66		4	120
4539	Adrenocortical adenoma	F	42		3,5	74
1151	Adrenocortical carcinoma	M	30	Yes	11	48
1739	Adrenocortical carcinoma	F	40		18	176
3072	Adrenocortical carcinoma	M	68	Yes	12	84
3353	Adrenocortical carcinoma	F	35		9	108
3410	Adrenocortical carcinoma	F	84	Yes	19	24^b^
372	Adrenocortical carcinoma	M	78	Yes	15	4^b^
2931	Adrenocortical carcinoma	M	52		11	123
882	Adrenocortical carcinoma	F	72	Yes	7	6^b^

a *Time between surgery and follow-up.*

b *Dead of disease.*

### Microsomal preparation

Microsomes were prepared essentially as described in[38]. Briefly, tissue samples were homogenized by initial crushing in a mortar with 1 mL 0.1 M potassium phosphate buffer, pH 7.4, 0.25 M sucrose and protease inhibitors (Complete Mini, Roche Diagnostics). The semi-crushed tissues were then transferred to eppendorf tubes and porcelain beads were added. The samples were put in a Retsch Mixer Mill (MM 301) and homogenized 2×30 seconds on the highest speed (30/s). Final homogenization was performed by probe sonication (on ice, 4×10 seconds, power: 30%, Bandelin Sonopuls, Buch & Holm). The homogenate was centrifuged at 10 000 rpm at 4°C for 10 minutes and the pellet was discarded. The protein suspensions were centrifuged at 100 000×g at 4°C for 1 hour. The resulting supernatants containing soluble proteins were stored at −20°C and the pellets were suspended in 500 µL 2.5 M NaBr for 45 min on ice with shaking. Another centrifugation was performed at 4°C for 1 hour at 100 000×g. The supernatant containing membrane-associated proteins and the pellets containing microsomes were stored separately at −20°C until further analysis.

### Digestion and iTRAQ labeling

The microsomal proteins were delipidated and precipitated according to the method described by Wessel and Flügge [39]. The protein pellets were solubilized in 50 µL 1% SDS, after which additional 50 µL water was added (final SDS concentration 0.5%). If full dissolvation was not reached, the samples were centrifuged at 10 000 rpm for 5 minutes and pellet discarded. An SDS-PAGE was run on the un-dissolved fractions and no proteins could be observed after coomassie staining (data not shown). The protein concentrations were measured using the Bio-Rad D_C_ protein assay (Bio-Rad Laboratories, Hercules, CA, USA). The samples were then diluted to 0.1% SDS using water and 1 M TEAB buffer (final concentration TEAB buffer 0.025 M). To 75 µg protein from each sample, DTT (final concentration 5 mM, 30 min at 56°C) and iodoacetamide (final concentration 0.015 M, 30 min, dark) was added. Trypsin (modified sequencing grade, Promega, Madison, WI, USA) was added (1∶50, trypsin:protein) and the samples were incubated at 37°C over night. An SDS-PAGE was run on undigested and digested sample to ensure satisfactory digestion (data not shown). A pooled internal standard was made by combining 15 µg from each sample. Subsequently, 50 µg of each sample were labeled and pooled using the 8-plex iTRAQ kit (Applied Biosystems, Foster City, CA, USA) according to the manufacturer's instructions (see Fig. 1 for experimental layout). Excess reagent was removed from the pooled sample using an SCX-cartridge (StrataSCX, Phenomenex, Torrence, CA, USA). The eluate was dried in a speed-vac.

### Isoelectric focusing of peptides

iTRAQ-labeled tryptic peptide samples were dissolved in 200 µL 8 M urea. Narrow range IPG-strips for peptide focusing (pH 3.7–4.9, 24 cm long) together with dry sample application gels (33×3×2 mm) were kindly supplied by GE Healthcare Bio-Sciences AB, Uppsala, Sweden. The application gels were rehydrated in sample over night while the strips were rehydrated over night in 8 M urea and 1% Pharmalyte™ 2.5–5 (GE Healthcare Bio-Sciences AB, Uppsala, Sweden). The IPG strips were put in the focusing tray and the application gels containing the samples were placed on the anodic end of the IPG strips with filter paper between the application gels and the electrodes. The strips were covered with mineral oil and the focusing was performed on an Ettan™ IPGphor™ (GE Healthcare Bio-Sciences AB, Uppsala, Sweden) until 100 kVh had been reached. After focusing, peptides were extracted from the strips by a prototype liquid handling robot, kindly supplied by GE Healthcare Bio-Sciences AB. A plastic device with 72 wells was put onto each strip and 50 µl of MQ water was added to each well. After 30 minutes incubation, the liquid was transferred to a 96 well plate and the extraction was repeated 2 more times. Samples were then freeze dried in SpeedVac and kept at −20°C. Prior to analysis, each fraction was re-suspended in 8 µl 3% acetonitrile and 0.1% formic acid.

### LC-ESI-LTQ-Orbitrap analyses

LC-MS was performed on a hybrid LTQ-Orbitrap Velos mass spectrometer (Thermo Fischer Scientific, San Jose, CA, USA). An Agilent HPLC 1200 system (Agilent Technologies, Santa Clara, CA, USA) was used for online reversed-phase nano-LC at a flow of 0.4 µl/min. Solvent A was 97% water, 3% ACN, 0.1% formic acid; and solvent B was 5% water, 95% ACN, 0.1% formic acid. The curved gradient went from 2% B up to 40% B in 45 min, followed by a steep increase to 100% B in 5 min. Samples (3 µl from each IPG fraction) were trapped on Zorbax 300SB-C18, 5 µm, 5×0.3 mm (Agilent Technologies, Santa Clara, CA, USA) and separated on a NTCC-360/100-5-153 C18 column (Nikkyo Technos Co., Tokyo, Japan) installed on to the nano electrospray ionisation (NSI) source of the Orbitrap Velos instrument. Acquisition proceeded in ∼3.5 s scan cycles, starting by a single full scan MS at 30000 resolution (profile mode), followed by two stages of data-dependent tandem MS (centroid mode): the top 5 ions from the full scan MS were selected firstly for collision induced dissociation (CID, at 35% energy) with MS/MS detection in the ion trap, and finally for high energy collision dissociation (HCD, at 50% energy) with MS/MS detection in the orbitrap. Precursors were isolated with a 2 m/z width and dynamic exclusion was used with 60 s duration.

### Data analysis

The MS/MS spectra were searched by Sequest combined with the Percolator algorithm (version 2.0) for PSM search optimization using Proteome Discoverer 1.3 (Thermo Fischer Scientific, San Jose, CA, USA) against the Swissprot protein sequence database (update 2012-02-02). A precursor mass tolerance of 10 ppm, and product mass tolerances of 0.02 Da for HCD-FTMS and 0.8 Da for CID-ITMS were used. Further settings used were: trypsin with 1 missed cleavage; carbamidomethylation on cysteine and iTRAQ-8plex on lysine and N-terminal as fixed modifications; and oxidation of methionine and phosphorylation on serine, tyrosine, threonine as variable modifications. Quantitation of iTRAQ-8plex reporter ions was done by Proteome Discoverer on HCD-FTMS tandem mass spectra using an integration window tolerance of 20 ppm. Results were limited to ≥1 high confident peptide (99%) using a false discovery rate of <1%.

Pathway analysis was carried out using Ingenuity Systems Pathway Analysis (IPA) program (Redwood City, CA, USA). Hierarchical clustering was carried out using GENE-E software (version 2.1.154, http://www.broadinstitute.org/cancer/software/GENE-E/). Enrichment analysis was done using ProteinCenter (Software Version 3.10.10004, Thermo Fischer Scientific, San Jose, CA, USA) and GOrilla [7], [8].

### Statistical analysis

Univariate data analysis was done in R by performing a student's t-test with correction for multiple testing (Benjamini-Hochberg FDR). Multivariate data analysis was carried out using the Simca-P+, version 12.0.0.0 (Umetrics AB, Umeå, Sweden). First, a Principal Component Analysis (PCA) was performed [40]. This is an unsupervised method that can be used to get an overview of the data and identify trends, groups or outliers. Next, an OPLS-DA (Orthogonal Projections to Latent Structures-Discriminant Analysis) was performed [41]. OPLS-DA is a supervised prediction method suitable when the number of variables (proteins) greatly exceeds the number of observations (samples). OPLS maximizes the covariance and correlation between x and y data (in this case x is the protein id and quantity and y is the tumor type). OPLS also removes structured noise in the x data that is orthogonal to the response, y. A predictive model was created based on the 1081 proteins and their quantitative information. The Variable Importance on Projection (VIP) scores were utilized for variable selection in an iterative process until a satisfactory model had been found.

### Western blot

Microsomal proteins (50 µg) were separated with SDS-PAGE and blotted on Hybond ECL™ nitrocellulose membranes (GE Healthcare Bio-Sciences AB, Uppsala, Sweden). The membranes were blocked with 5% non-fat milk in TRIS-buffered saline (TBS)/0.05% Tween for 1 h and then probed with primary antibody (anti-ALDOA, dilution 1∶500, HPA004177, Atlas Antibodies AB, Stockholm, Sweden; anti-SHMT2, dilution 1∶500 HPA020543, Atlas Antibodies AB, Stockholm, Sweden; anti-NDUFA13 (GRIM-19), dilution 1∶750, H00051079-B02P, Abnova Corporation, Taipei, Taiwan) diluted in 2.5% non-fat milk in TBS/Tween overnight at 4°C. The immunoreaction was visualized using horseradish peroxidase-conjugated sheep anti-mouse IgG or donkey anti-rabbit (GE Healthcare Bio-Sciences AB, Uppsala, Sweden) diluted in 2.5% non-fat milk in TBS/Tween for 1 h in room temperature, followed by use of SuperSignal West Pico Chemiluminescent Substrate (Pierce Biotechnology Inc, Rockford, IL, USA).

### Immunohistochemistry

Paraffin samples of 3 benign and 4 malignant adrenal tumors and control tissue samples were available for analysis. The tissue was fixed routinely in 4% formaldehyde buffered solution and paraffin embedded. All adrenal tumors were studied by routine histochemical staining i.e. haematoxylin and eosin staining. To perform immunohistochemical analysis tissue sections were cut at 4 µm, deparaffinized and rehydrated. In our experience, antigen retrieval by heating in citrate buffer was necessary to obtain a distinct signal without interfering background. All antibodies were tested at different dilution with and without antigen retrieval technique. As positive controls, tissue samples from pancreatic gland (NDUFA13/GRIM-19) and normal adrenal gland (STAT-3) were used. Negative controls were performed by replacing the primary antibody with phosphate buffer. The sections were incubated in 0.3% hydrogen peroxide in water for 30 min, blocked in 1% BSA with 0.01% sodium azide for 45 min, and incubated with primary antibody diluted in 1% BSA overnight using concentrations determined from dilution trials with positive controls as follows: NDUFA13 ( = GRIM-19), dilution 1∶100, mouse polyclonal affinity purified antibody raised against a human full-length 144 amino acid sequence, H00051079-B02P, Abnova Corporation, Taipei, Taiwan; STAT-3, dilution 1∶200, rabbit polyclonal affinity purified raised against a human 149 amino acid sequence, HPA001671, Atlas Antibodies AB. The antigen-antibody binding site was visualized using the avidin-biotin complex method (Vectastain Elite kit, Vector Laboratories, Burlingame, CA), colour reaction using diaminobenzidine tetrahydrochloride (DAB) and counter stained with haematoxylin.

## Supporting Information

Fig. S1 Immunohistochemistry analysis regarding GRIM-19 and STAT3 expression. GRIM-19 expression was evaluated in three benign and three malignant tumor tissues. STAT3 expression was evaluated in two benign and two malignant tumor tissues.
(JPG)

Table S1 All proteins that were identified in the iTRAQ experiments.
(XLSX)

Table S2 All proteins that were quantified in the iTRAQ experiments.
(XLSX)

Table S3 The proteins that overlapped in the univariate and multivariate data analyses.
(DOCX)

Table S4 Gene names and corresponding protein names of the proteins depicted in Fig.6.
(DOCX)

Table S5 Protein and peptide identification statistics for iTRAQ experiment 1.
(XLSX)

Table S6 Protein and peptide identification statistics for iTRAQ experiment 2.
(XLSX)
