# Supplementary figures and images for: Differentially Expressed Proteins in Malignant and Benign Adrenocortical Tumors
Source: PLoS One. 2014 Feb 3;9(2):e87951. doi: 10.1371/journal.pone.0087951 (PMC3912167; doi:10.1371/journal.pone.0087951)

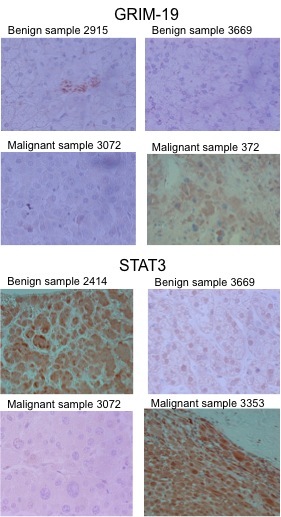

Supplement: Fig. S1 — Immunohistochemistry analysis regarding GRIM-19 and STAT3 expression. GRIM-19 expression was evaluated in three benign and three malignant tumor tissues. STAT3 expression was evaluated in two benign and two malignant tumor tissues. (JPG) [file pone.0087951.s001.jpg]
