# Supplementary material for: Differentially Expressed Proteins in Malignant and Benign Adrenocortical Tumors
Source: PLoS One. 2014 Feb 3;9(2):e87951. doi: 10.1371/journal.pone.0087951 (PMC3912167; doi:10.1371/journal.pone.0087951)
Supplement: Table S4 — Gene names and corresponding protein names of the proteins depicted in Fig.6. (DOCX) [file pone.0087951.s005.docx]

Table S4. Accession numbers, gene names and descriptions of the proteins depicted in Fig. 6

| **Accession** | **Gene name** | **Protein description** |
| --- | --- | --- |
| O43505 | B3GNT1 | N-acetyllactosaminide beta-1,3-N-acetylglucosaminyltransferase OS=Homo sapiens GN=B3GNT1 PE=1 SV=1 - [B3GN1_HUMAN] |
| O43674 | NDUFB5 | NADH dehydrogenase [ubiquinone] 1 beta subcomplex subunit 5, mitochondrial OS=Homo sapiens GN=NDUFB5 PE=1 SV=1 - [NDUB5_HUMAN] |
| O43676 | NDUFB3 | NADH dehydrogenase [ubiquinone] 1 beta subcomplex subunit 3 OS=Homo sapiens GN=NDUFB3 PE=1 SV=3 - [NDUB3_HUMAN] |
| O43677 | NDUFC1 | NADH dehydrogenase [ubiquinone] 1 subunit C1, mitochondrial OS=Homo sapiens GN=NDUFC1 PE=2 SV=1 - [NDUC1_HUMAN] |
| O76062 | TM7SF2 | Isoform 2 of Delta(14)-sterol reductase OS=Homo sapiens GN=TM7SF2 - [ERG24_HUMAN] |
| O95298 | NDUFC2 | NADH dehydrogenase [ubiquinone] 1 subunit C2 OS=Homo sapiens GN=NDUFC2 PE=1 SV=1 - [NDUC2_HUMAN] |
| O95299 | NDUFA10 | NADH dehydrogenase [ubiquinone] 1 alpha subcomplex subunit 10, mitochondrial OS=Homo sapiens GN=NDUFA10 PE=1 SV=1 - [NDUAA_HUMAN] |
| P05093 | CYP17A1 | Steroid 17-alpha-hydroxylase/17,20 lyase OS=Homo sapiens GN=CYP17A1 PE=1 SV=1 - [CP17A_HUMAN] |
| P07099 | EPHX1 | Epoxide hydrolase 1 OS=Homo sapiens GN=EPHX1 PE=1 SV=1 - [HYEP_HUMAN] |
| P07237 | P4HB | Protein disulfide-isomerase OS=Homo sapiens GN=P4HB PE=1 SV=3 - [PDIA1_HUMAN] |
| P36021 | SLC16A2 | Monocarboxylate transporter 8 OS=Homo sapiens GN=SLC16A2 PE=1 SV=2 - [MOT8_HUMAN] |
| P49821 | NDUFV1 | Isoform 2 of NADH dehydrogenase [ubiquinone] flavoprotein 1, mitochondrial OS=Homo sapiens GN=NDUFV1 - [NDUV1_HUMAN] |
| P51648 | ALDH3A2 | Fatty aldehyde dehydrogenase OS=Homo sapiens GN=ALDH3A2 PE=1 SV=1 - [AL3A2_HUMAN] |
| Q01082 | SPTBN1 | Spectrin beta chain, brain 1 OS=Homo sapiens GN=SPTBN1 PE=1 SV=2 - [SPTB2_HUMAN] |
| Q06278 | AOX1 | Aldehyde oxidase OS=Homo sapiens GN=AOX1 PE=2 SV=2 - [ADO_HUMAN] |
| Q13813 | SPTAN1 | Isoform 3 of Spectrin alpha chain, brain OS=Homo sapiens GN=SPTAN1 - [SPTA2_HUMAN] |
| Q6PIU2 | NCEH1 | Isoform 3 of Neutral cholesterol ester hydrolase 1 OS=Homo sapiens GN=NCEH1 - [NCEH1_HUMAN] |
| Q6UWH4 | FAM198B | Protein FAM198B OS=Homo sapiens GN=FAM198B PE=2 SV=1 - [F198B_HUMAN] |
| Q8NFH4 | NUP37 | Nucleoporin Nup37 OS=Homo sapiens GN=NUP37 PE=1 SV=1 - [NUP37_HUMAN] |
| Q96AG4 | LRRC59 | Leucine-rich repeat-containing protein 59 OS=Homo sapiens GN=LRRC59 PE=1 SV=1 - [LRC59_HUMAN] |
| Q96LZ7 | FAM82A1 | Isoform 4 of Regulator of microtubule dynamics protein 2 OS=Homo sapiens GN=FAM82A1 - [RMD2_HUMAN] |
| Q9BVL2 | NUPL1 | Isoform 2 of Nucleoporin p58/p45 OS=Homo sapiens GN=NUPL1 - [NUPL1_HUMAN] |
| Q9H6X2 | ANTXR1 | Isoform 3 of Anthrax toxin receptor 1 OS=Homo sapiens GN=ANTXR1 - [ANTR1_HUMAN] |
| Q9NXW2 | DNAJB12 | DnaJ homolog subfamily B member 12 OS=Homo sapiens GN=DNAJB12 PE=1 SV=4 - [DJB12_HUMAN] |
| Q9P0J0 | NDUFA13 | NADH dehydrogenase [ubiquinone] 1 alpha subcomplex subunit 13 OS=Homo sapiens GN=NDUFA13 PE=1 SV=3 - [NDUAD_HUMAN] |
| Q9P2W9 | STX18 | Syntaxin-18 OS=Homo sapiens GN=STX18 PE=1 SV=1 - [STX18_HUMAN] |
